# COVID-19 related visiting ban in nursing homes as a source of concern for residents’ family members: a cross sectional study

**DOI:** 10.1186/s12912-022-01036-4

**Published:** 2022-09-14

**Authors:** Jari Pirhonen, Leena Forma, Ilkka Pietilä

**Affiliations:** 1 University of Helsinki, Po. Box 9, 00014 Helsinki, Finland; 2 Laurea University of Applied Sciences, Ratatie 22, 01300 Vantaa, Finland

**Keywords:** Concern, COVID-19, Family members, Information delivery, Long-term care, Wellbeing

## Abstract

**Background:**

Visiting a close relative who resides in a nursing home is an opportunity for family members to extend their caring roles and find reassurance that the older person’s life is continuing as well as possible. At the same time, visits allow family members to observe the quality of formal care in the facility. In Finland, the COVID-19 pandemic led to the imposition of visiting bans in nursing homes in March 2020, thereby preventing customary interaction between residents and their family members. The aim of this study is to investigate family members’ experiences of the visiting ban and its effects on their concern over the wellbeing of close relatives living in nursing homes.

**Methods:**

A cross-sectional study was conducted to explore family members’ self-reported concerns and the factors associated with those concerns. In the context of this unpredictable pandemic, this was considered an appropriate approach, as information at the very beginning of the visiting ban was sought, and causal relations were not investigated. The data consist of a quantitative survey (*n* = 366) conducted among family members in May–June 2020. Binary logistic regression analyses were performed to explore the association between the independent variables and reported concern.

**Results:**

The results showed that increased concern was extremely common (79%). The factors associated with this notable increase were adequacy of contact and information, observations of changes in the wellbeing of the relative in question, and doubts over the appropriateness of the visiting restriction.

**Conclusions:**

In light of the findings, care providers should improve their information provision to residents’ family members and find new ways of allowing visits to nursing homes in the future in all circumstances.

## Introduction

The COVID-19 pandemic spread quickly across Europe in March 2020 [1, 2]. In Finland, an Emergency Powers Act came into effect on 17 March [3], which strongly affected social interaction among citizens: schools were closed, people were urged to telework, the Capital Region of Helsinki was locked down, restaurants were shut, and public events were cancelled. It was assumed that the virus was especially dangerous for older adults [4]; therefore, people over the age of 70 were urged to stay at home in quarantine-like conditions. As one way of safeguarding the health of frail older adults, the Act banned visits to nursing homes by residents’ family members (FM). In Finland, as in many other countries, these restrictive rules also applied to professionals who were not part of the regular nursing staff, such as physiotherapists and hairdressers [5], thus effectively ending the social and leisure-time activities of residents [5, 6]. The strict visiting ban probably saved lives, given that the virus has proved to be particularly lethal to residents of nursing homes. Fewer than one percent of Finnish citizens reside in these homes, yet 44 percent of all COVID-19-related deaths occurred in them during the first months of the pandemic [7]. This high mortality rate mirrors the situation on the international level [8, 9].

Although the Finnish Emergency Powers Act was repealed on 16 June 2020, and many social restrictions were lifted during the summer, visiting in nursing homes was not normalized. Visits were allowed outdoors, and sometimes in specific meeting spaces constructed inside the facilities, but FMs were only permitted to enter residents’ private dwellings under exceptional circumstances, such as in cases of terminal care. In early autumn, visits to residents’ rooms were allowed, albeit with the imposition of strict safety measures, but in late 2020 the situation worsened again with the spread of the second wave of the virus [10–12]. Visiting restrictions were tightened once more. The same development occurred in autumn 2021. In August, the situation improved, but in December the Omicron variant cast doubt on the future [13]. In other words, FMs were unable to visit nursing home facilities freely for almost two years, and some safety measures, such as the recommendation on the use of face masks, still apply in spring 2022.

There are some 50,000 residents in Finnish nursing homes [14], thus, including FMs, the visiting ban affects the lives of hundreds of thousands of people. Previous research has shown that visits to care facilities are important to both residents and FMs [15–18]. FM visits promote residents’ self-esteem [19] and sense of autonomy [15] and relieve psychological stress [20]. Moreover, FMs provide social and emotional support and alleviate feelings of loneliness [18, 21]. In many cases, FMs help residents with personal, instrumental, relational, and recreational matters during their visits [22], and they are in a unique position in terms of understanding, articulating, and supporting the emotional, social, and health needs of those who are frail [6]. They also help residents maintain their identities [16], which is extremely important, since memory disorders are the primary reason for entering nursing homes [23], and therefore the vast majority of residents suffer from various forms of dementia [24].

The individuals who are most likely to visit nursing homes are female FMs, including wives, daughters, and daughters‐in‐law [25, 26]. Previous research shows that the more often FMs visit their close relative, the more involved they become in their care [22]. In addition to offering support, FMs also rely on their visits to alleviate their own concerns: they can rest assured that their relatives are living their lives as well as they can [16, 27]. On the other hand, FMs may participate in the caring process because they believe that the quality of care provided is closely connected to their level of involvement [27]. As Hertzberg and Ekman [28] found, FMs believe that residents are often left alone and inactive for long periods of time. In another study [29], they concluded that FMs were content with the physical care but were doubtful about the quality of psychological care. Moreover, a survey conducted by Vohra et al. [30] revealed FMs’ low satisfaction with staffing levels, updating practices, and the involvement of families in care planning and decision-making. Thus, one reason for FMs’ frequent visits could relate to their mistrust of the quality of care.

In this context, mistrust arises from poor communication between FMs and nursing staff [31]. According to Hertzberg and Ekman [28], FMs feel that they have received incorrect or insufficient information and, in their opinion, have been misled into making wrong decisions on behalf of themselves or their relative. Majerovitz, Mollott, and Rudder [32] also found specific communication problems identified by FMs, including shaming practices, criticism of their involvement, lack of information, changes without consultation, the staff’s lack of sufficient time to talk, high staff turnover, rotating shifts, and poor intra-staff communication. Furthermore, daughters and daughters‐in‐law, who tend to be the most frequent visitors to care facilities, report poorer communication with staff than do other FMs [33]. In addition, rapid privatization of long-term elderly care [34] has raised doubts and questions in public debate. The year 2018 was disastrous for public perceptions of elderly care in Finland, as the supervising authorities were forced to close several privately owned nursing homes due to their neglect of current rules on staffing ratios and the poor quality of medication. In Finland and Sweden, rapid, widespread privatization of care has compromised the very basis of the Nordic welfare state, i.e., strong universalism in health and social services [35]. In such a cultural atmosphere, one potential reason for FMs’ concerns is their perception of the inability of eldercare facilities to provide quality care for their elderly relatives.

Ultimately, nursing home care is a socially constructed process [36, 37], and therefore quality care also acknowledges residents’ relatives [17, 18]. The first reports on visiting bans resulting from the COVID-19 pandemic indicate negative effects on the wellbeing of residents in nursing homes [38, 39]. In particular, such bans represent a challenge to residents’ psychological well-being [40, 41]. These early findings are now being complemented by a growing number of studies on the issue since the doors of nursing homes have opened to researchers after the lockdown. However, as noted above, visits also affect the wellbeing of FMs [16, 27]. Nonetheless, far less knowledge exists on their visiting-ban-related wellbeing. A search of the PubMed, Cinahl, and Medline databases reveals a small number of recent studies on the subject. For example, Ting-Chun et al. [42] found that the better the visiting restrictions were accepted, the less worries were reported by FMs. In turn, research by Paananen et al. [43] revealed that FMs feared that their relatives in nursing homes would fade away both emotionally and physiologically during the visiting ban. In addition, the loss of their caregiving role has proven to be a major source of distress to family members [44].

This exploratory study investigates family members’ concern over relatives residing in nursing homes and the factors associated with the concern. We seek answers to two distinct research questions: (i) to what extent did the visiting restrictions of spring 2020 cause changes in levels of concern and wellbeing among the FMs of nursing home residents? (ii) Which factors were associated with increased concern among FMs?

## Data and methods

### Context

We conducted a cross-sectional study, which, in this unpredictable pandemic situation, was considered an appropriate approach, as we sought information at the very beginning of the visiting ban and were not interested in causal relations. An online questionnaire was created to capture FMs’ experiences of the visiting ban: it comprised seven questions on the backgrounds of the respondents and 17 multiple-choice questions on the research topic. Since the COVID-19 situation was sudden and the duration of the pandemic unpredictable, it was necessary to create the questionnaire quickly. Therefore, we chose not to pre-test it with our target population; to compensate, we nonetheless involved several researchers in its drafting process.

The questionnaire was created with a SurveyHero tool and distributed primarily via Facebook and Twitter. On Facebook, it was shared specifically with groups linked to elderly care. On Twitter, the call to participate was re-tweeted 41 times. In addition, two major Finnish non-profit organizations advancing the wellbeing of older people promoted the survey on their webpages and bulletins. The survey was launched on 11 May and closed on 30 June 2020.

### Variables

The dependent variables were the wellbeing of the FM and concern among FMs over the wellbeing of a close relative residing in a nursing home during the visiting ban. All the questions used in this study are described in Table 1.

**Table 1:** Description of variables: the questions and answer options

Question	Answer options
**Dependent variables**	
Have you been so concerned about the wellbeing of your close one that your own wellbeing had deteriorated before the visiting ban?	Yes, I have
	No, I have not
Have you been so concerned about the wellbeing of your close one that your own wellbeing had deteriorated during the visiting ban?	Yes, I have
	No, I have not
Compared to the time before the visiting ban, has your concern about the wellbeing of your close one during the visiting ban..?	Remained similar
	Decreased
	Increased to some extent
	Increased notably
	No concern during the whole time in nursing home
**Independent variables**	
**Background information**	
What is your age?	(free space)
What is your gender?	Woman
	Man
	Other
What is your relation to person living in a nursing home?	S/he is my
	Spouse
	Mother or father
	Sibling
	Other, what?
How long has your close one lived in a nursing home?	< 3 months
	3–6 months
	6 months – 1 year
	years
	> 2 years
How long is the distance from your home to the nursing home?	< 1 km
	1–5 km
	5–10 km
	10–20 km
	< 20 km, how long?
**Before the visiting ban**	
How often did you visit your close one in the nursing home before the visiting ban?	Almost every day
	At least twice a week
	About once a week
	Every second week
	Once a month
	Less than once a month
Have you been happy with the frequency of your visits before the visiting ban?	Yes, I have
	No, I visited too often
	No, I visited too rarely
**During the visiting ban**	
Do you feel that you are able to have enough contacts with your close one during the visiting ban?	Yes, I do
	No, I do not
Have you noticed changes in wellbeing of your close one during the visiting ban?	Yes, I have
	No, I have not
Have you received enough information from the staff of nursing home on	
Wellbeing of close on	Yes / No
Safety of close one	Yes / No
Daily life of close one	Yes / No
Possibilities to keep contact with close one	Yes / No
Changes in daily life of nursing home	Yes / No
Do you think that restricting the visits of family members to nursing homes during the corona epidemic is the right solution?	Yes, I do
	No, I do not

The survey contained two items on the wellbeing of relatives: “Have you been so concerned about the wellbeing of your close relative living in a nursing home that your own wellbeing has decreased 1) before the visiting ban and 2) during the visiting ban?” The answer was “yes, I have,” or “no, I have not.”

The survey item inquiring about the concern was “compared to the time before the visiting ban, has your concern about the wellbeing of your close relative living in a nursing home 1) stayed at the same level, 2) increased notably, 3) increased to some extent, 4) decreased? or 5) I have not been concerned during the time my relative has been residing in a nursing home.” We used this five-point scale in the preliminary analyses. In turn, we coded the variable for the binary logistic regression analyses as follows: 1 = increased notably and 0 = remained similar, decreased, no concern, and increased to some extent. We chose a notable increase in concern as the dependent variable given that people tend to choose the middle option (central tendency bias: [45]), and we wished to highlight a specific group of respondents choosing the extreme option.

The independent variables represent the background information and the situation prior to and experiences during the visiting ban (Table 1). Age was collected as a continuous variable but subsequently classified in five categories (< 40, 40–49, 50–59, 60–69, 70 + years). Three options were provided concerning the relationship of the respondent to the person living in a nursing home: spouse, parent, or sibling. From the free-space answers, we added grandparent and parent-in-law, which transpired to be rather large groups, and classified the rest as “other.” The relationship is presented as a characteristic of the respondent, not of the nursing-home resident. Distance from the facility was classified in three groups of equal size (< 5 km, 5–20 km, > 20 km).

The question on receiving information during the visiting ban included five aspects: “did you receive enough information about your close relative concerning 1) wellbeing, 2) safety, 3) daily life, 4) being able to keep in contact, and 5) changes in daily life?” The response options were “yes” or “no.” We coded a sum variable to describe receiving sufficient information in general (1 = sufficient information on 4 or 5 issues, 0 = sufficient information on 0–-3 issues). Both the original responses and the sum variable are described and used in the preliminary analyses, but we only included the sum variable in the binary logistic regression analyses.

### Analyses

Given that all the variables were categorical, we present the frequencies and the percentages. We tested the associations of between the independent variables and the dependent variables using cross tabulation and Chi square tests, having excluded respondents with missing observations in each variable from these preliminary analyses.

We then performed binary logistic regression analyses to explore the association between the independent variables and a notable increase in concern. We ran three models:

- Model 1: variables describing background information,
- Model 2: model 1 + variables describing the situation before the visiting ban, and
- Model 3: model 2 + variables describing experiences during the visiting ban.

The analyses were performed using IBM SPSS Statistics (v. 28.0).

### Research ethics

The Ethical Review Board in the Humanities and Social and Behavioural Science, located at the University of Helsinki, was consulted about the need for ethical approval for the research, and the committee confirmed that it was unnecessary to apply for ethical approval. While no external ethical evaluation was required for this survey study, data were collected in accordance with the General Data Protection Regulation of European Union (GDPR, EU 2016/679) and Finnish research ethical principles (https://tenk.fi/en). Participants were informed about data management on the first page of the survey. Currently, the dataset is being used in several academic projects, but it will be deleted once these projects have been finalized.

## Results

### Background information

A total of 410 persons responded to the online questionnaire, of whom 42 returned it incomplete. Moreover, one response concerned a person who had died many years ago, and another contained missing information concerning one of the dependent variables. After excluding these responses, the dataset comprised answers from 366 participants. In addition, for the regression analyses, we excluded all respondents with missing observations in any of the independent variables (*n* = 329). The university of (Anonymized) owns the data.

Of the respondents, 88 percent were female (Table 2). The most common age group was 50–59 years, and most of the respondents were offspring of the person living in the nursing home. Most of the residents had lived in the nursing home more than a year, 46 percent for more than two years. The distance from home to the nursing home was less than 20 km for 66 percent of the respondents, and 67 percent had visited their relatives at least once a week before the ban on visits. The majority of respondents were happy with the frequency of their visits, although 19 percent replied that they visited too rarely.

**Table 2:** Basic characteristics (*n* = 366)

	N	%
**Background information**		
Age group		
< 40	34	9.3
40–49	56	15.3
50–59	125	34.2
60–69	95	26.0
70 +	42	11.5
Missing observations	14	3.8
Gender		
Female	323	88.3
Male	41	11.2
Missing observations	2	0.5
Relation to person living in a nursing home		
Spouse	38	10.4
Child	243	66.4
Sibling	14	3.8
Grandchild	27	7.4
Child-in-law	14	3.8
Other	30	8.2
Length of residence in a nursing home		
< 3 months	35	9.6
3–6 months	39	10.7
6 months – 1 year	44	12.0
1-2 years	80	21.9
> 2 years	168	45.9
Distance from the nursing home		
< 5 km	124	33.9
5–20 km	117	32.0
> 20 km	125	34.2
**Before the visiting ban**		
Frequency of visits to the nursing home		
Almost every day	51	13.9
At least twice a week	86	23.5
About once a week	108	29.5
Every second week	53	14.5
Once a month	41	11.2
Less than once a month	20	5.5
Missing observations	7	1.9
Happy with the frequency of visits before the visiting ban		
Yes	285	77.9
Too often	3	0.8
Too rarely	69	18.9
Missing observations	9	2.5
So concerned about the wellbeing of a close one that their own wellbeing had deteriorated before the visiting ban		
Yes	81	22.1
No	282	77.0
Missing observations	3	0.8
**During the visiting ban**		
Enough contacts during the visiting ban		
Yes	86	23.5
No	279	76.2
Missing observations	1	0.3
Noticed changes in wellbeing of the close one		
Yes	225	61.5
No	132	36.1
Missing observations	9	2.5
Received enough information on Wellbeing		
Yes	236	64.5
No	117	32.0
Missing observations	13	3.6
Safety		
Yes	207	56.4
No	146	39.8
Missing observations	14	3.8
Daily life		
Yes	192	52.3
No	164	44.7
Missing observations	11	3.0
Possibilities to keep contact		
Yes	208	56.7
No	148	40.3
Missing information	11	3.0
Changes in daily life		
Yes	154	42.0
No	197	53.7
Missing observations	16	4.4
Sum variable: enough information ^a^		
Yes (on 4–5 issues)	147	42.7
No (on 0–3 issues)	197	57.3
Concern about the wellbeing of a close one during the visiting ban		
No concern at all	19	5.2
Remained similar	50	13.7
Decreased	7	1.9
Increased to some extent	137	37.4
Increased notably	153	41.8
So concerned about the wellbeing of a close one that their own wellbeing had deteriorated during the visiting ban		
Yes	170	46.4
No	194	53.0
Missing observations	2	0.5
Visiting restrictions are the right solution		
Agree	198	54.1
Do not agree	162	44.3
Missing observations	6	1.6

^a^ *n* = 344, missing observations in original variables excluded

Seventy-six percent of respondents felt that they lacked sufficient contact with their relatives while the visiting ban was in place (Table 2). Moreover, 62 percent reported having noticed changes in the wellbeing of their relative, and 32–54 percent felt that they failed to receive sufficient information from the nursing home. In terms of the adequacy of information, the highest level of satisfaction concerned information on the wellbeing of the relative, while the lowest level related to changes in daily life during the pandemic. More than half (54%) considered that restricting visits due to coronavirus was the right solution.

### Concern and wellbeing among relatives

Twenty-two percent of respondents reported that they were so concerned about the wellbeing of their relatives living in a nursing home that their own wellbeing had deteriorated (Table 2).

By contrast, half (49%) of the respondents reported no adverse effects on their wellbeing due to concern before or during the ban. In turn, almost one fifth (19%) reported a decrease in wellbeing both before and during the ban, whereas 27 percent reported adverse effects during the ban but not prior to its imposition (figures not shown).

**Fig. 1:**
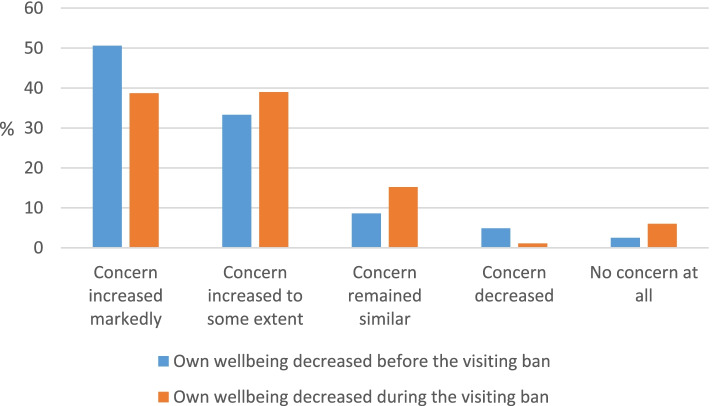
Change in concern among family members during the visiting ban. Legend: Being so concerned about the wellbeing of a relative that their own wellbeing deteriorated before / during the ban, *N* = 364

As many as 79 percent of respondents reported increased concern about the wellbeing of their relatives during the visiting ban: 37 percent to some extent, and 42 percent markedly (Table 2). Moreover, 46 percent reported being so concerned that their own wellbeing had been adversely affected (Table 2, Fig. 1).

Fifty-one percent of those whose own wellbeing had deteriorated before the ban due to concern reported a notable increase in concern during the ban, compared with 69 percent of those whose wellbeing had deteriorated during the ban (Table 3).

**Table 3:** Variables by concern about the wellbeing of a close one (%) during the visiting ban

	No concern	Remained similar	Decreased	Increased to some extent	Increased notably	*P*-value
**Background information**						
Age group, *n* = 352						
< 40	8.8	5.9	2.9	44.1	38.2	.670
40–49	3.6	8.9	1.8	35.7	50.0	
50–59	5.6	13.6	0.0	39.2	41.6	
60–69	5.3	13.7	4.2	36.8	40.0	
70 +	2.4	21.4	2.4	35.7	38.1	
Gender, *n* = 364						
Female	5.0	12.1	1.5	37.5	44.0	.058
Male	7.3	24.4	4.9	36.6	26.8	
Relation to person living in a nursing home, *n* = 366						
Spouse	2.6	13.2	2.6	31.6	50.0	.855
Child	5.3	13.6	2.1	35.8	43.2	
Sibling	0.0	28.6	0.0	35.7	35.7	
Grandchild	7.4	11.1	0.0	59.3	22.2	
Child-in-law	7.1	7.1	0.0	42.9	42.9	
Other	6.7	13.3	3.3	36.7	40.0	
Length of residence in a nursing home, *n* = 366						
< 3 months	2.9	22.9	8.6	17.1	48.6	.028
3–6 months	7.7	5.1	5.1	46.2	35.9	
6 months – 1 year	9.1	18.2	0.0	34.1	38.6	
1–2 years	6.3	10.0	0.0	37.5	46.3	
> 2 years	3.6	14.3	1.2	40.5	40.5	
Distance from the nursing home, *n* = 366						
< 5 km	4.0	12.1	1.6	37.1	45.2	.597
5–20 km	6.0	18.8	2.6	32.5	40.2	
> 20 km	5.6	10.4	1.6	42.4	40.0	
**Before the visiting ban**						
Frequency of visits to the nursing home, *n* = 359						
Almost every day	3.9	11.8	2.0	19.6	62.7	.012
At least twice a week	5.8	11.6	0.0	39.5	43.0	
About once a week	2.8	16.7	2.8	39.8	38.0	
Every second week	9.4	15.1	0.0	32.1	43.4	
Once a month	4.9	14.6	0.0	56.1	24.4	
Less than once a month	10.0	5.0	10.0	45.0	30.0	
Happy with the frequency of visits before the visiting ban, *n* = 357						
Yes	4.6	15.1	0.7	38.0	41.5	.122
Too often	33.3	0.0	0.0	33.3	33.3	
Too rarely	7.2	8.7	4.3	39.1	40.6	
So concerned about the wellbeing of a close one that their wellbeing had deteriorated before the visiting ban, *n* = 363						
Yes	2.5	8.6	4.9	33.3	50.6	.025
No	6.0	15.2	1.1	39.0	38.7	
**During the visiting ban**						
Enough contacts during the visiting ban, *n* = 365						
Yes	14.0	30.2	4.7	40.7	10.5	< .001
No	2.5	8.6	1.1	36.2	51.6	
Noticed changes in the wellbeing of a close one, *n* = 357						
Yes	2.2	6.2	2.2	34.2	55.1	< .001
No	10.6	27.3	1.5	43.2	17.4	
Received enough information on the wellbeing of a close one, *n* = 352						
Yes	6.8	19.6	2.6	40.9	30.2	< .001
No	1.7	3.4	0.9	29.1	65.0	
Safety, *n* = 352						
Yes	7.8	21.8	2.4	42.7	25.2	< .001
No	0.7	3.4	1.4	29.5	65.1	
Daily life, *n* = 355						
Yes	8.4	22.0	2.1	42.4	25.1	< .001
No	0.6	4.9	1.8	30.5	62.2	
Possibilities to keep in touch, *n* = 355						
Yes	7.2	21.2	1.9	38.5	31.3	< .001
No	2.0	4.1	2.0	34.0	57.8	
Changes in daily life, *n* = 350						
Yes	9.1	22.7	1.9	39.6	26.6	< .001
No	1.5	6.6	2.0	34.2	55.6	
Sum variable: enough information, *n* = 343						
For 4–5 issues	8.8	26.5	2.0	38.8	23.8	< .001
For 0–3 issues	1.5	4.6	2.0	35.7	56.1	
So concerned about the wellbeing of the close one that their own wellbeing had deteriorated during the visiting ban, *n* = 364						
Yes	1.2	2.4	0.6	26.5	69.4	< .001
No	8.8	23.7	3.1	47.4	17.0	
Visiting restrictions are the right solution, *n* = 360						
Agree	8.1	20.7	1.5	47.0	22.7	< .001
Do not agree	1.9	5.6	2.5	24.7	65.4	

*P*-values for Chi square tests; missing observations excluded from the analyses

### Participant characteristics by change in concern

We found a statistically significant association between the time relatives had lived in the nursing home and concern about their wellbeing, but the pattern was nonetheless opaque (Table 3). Visit frequency was also associated with concern: more frequent visitors were more likely to report a notable increase in concern, whereas concern among those who visited less frequently increased only to some extent.

All the variables describing experiences during the visiting ban were statistically significantly associated with the change in concern (Table 3). A larger proportion of those who reported noticing changes in wellbeing among their relatives also reported a notable increase in concern, as did 56–65 percent of those who failed to receive sufficient information; 40–43 percent of those who received inadequate information reported some increase in concern. Of those who disagreed with the visiting restrictions, 90 percent reported being increasingly concerned.

### Factors associated with a notable increase in concern

According to the results of the multivariate analyses, grandchildren were much less likely to report a notable increase in concern than were spouses (Table 4), a finding which was similar in all the models. No other variables describing background information were statistically significantly associated with a notable increase in concern.

**Table 4:** Factors associated with a notable increase in concern during the visiting ban, *N* = 329

	Model 1	*P*	Model 2	*P*	Model 3	*P*
	OR		OR		OR	
Constant	6.02	.039	6.55	0.04	2.45	.425
**Background information**						
Age (ref. < 40)						
40–49	1.05	.916	1.08	.880	1.38	.606
50–59	0.56	.221	0.58	.274	0.63	.451
60–69	0.45	.109	0.40	.081	0.41	.165
70 +	0.28	.066	0.27	.069	0.28	.153
Gender (ref. female)						
Male	0.46	.051	.52	.110	0.70	.461
Relation (ref. spouse)						
Child	0.41	.135	0.76	.665	0.42	.259
Sibling	0.29	.129	0.56	.511	0.48	.488
Grandchild	**0.08**	.002	**0.18**	.042	**0.07**	.011
Child-in-law	0.32	.160	0.68	.655	0.27	.183
Other	0.32	.098	0.55	.422	0.38	.294
Length of residence in a nursing home (ref. < 3 months)						
3–6 months	0.36	.085	0.41	.145	0.40	.202
6 months – 1 year	0.55	.280	0.77	.645	0.87	.839
1–2 years	0.86	.760	1.15	.791	1.20	.763
> 2 years	0.70	.444	1.02	.967	1.34	.615
Distance from the nursing home (ref. < 5 km)						
5–20 km	0.74	.297	0.74	.325	0.76	.453
> 20 km	0.84	.535	1.33	.393	1.36	.442
**Before the visiting ban**						
Frequency of visits to home care (ref. almost every day)						
At least twice a week			**0.38**	.022	0.41	.085
About once a week			**0.27**	.002	0.39	.077
Every second week			**0.25**	.009	0.35	.112
Once a month			**0.07**	< .001	**0.10**	.002
Less than once a month			**0.18**	.016	0.32	.170
Happy with the frequency of visits before the visiting ban (ref. yes)						
Too often			0.44	.560	0.32	.502
Too rarely			1.55	.223	1.12	.793
**During the visiting ban**						
Enough contacts during the visiting ban (ref. yes)						
No					**4.39**	.002
Noticed changes in the wellbeing of the close one (ref. yes)						
No					**0.32**	.001
Enough information on (ref. 0–3 issues)						
4–5 issues					**0.35**	.001
Visiting restrictions are the right solution (ref. yes)						
No					**3.23**	< .001
**Model statistics**						
Nagelkerke *R*^2^	0.09		0.16		0.46	

Dependent variable: 1 = increased notably, 0 = remained similar, decreased, no concern and increased to some extent. Binary logistic regression analyses, odds ratios (OR) and *p*-values

Those who visited close relatives living in a nursing home less frequently than “almost every day” were less likely to report a notable increase in concern (Table 4, Model 2), and the likelihood of such an increase fell gradually as the frequency of visits decreased. However, the association was no longer statistically significant when the variables describing the situation during the visiting ban were added to the model (Model 3). The only exception related to those who visited once a month.

Respondents who felt that they lacked sufficient contact with their close relatives during the visiting ban were several times more likely to report a notable increase in concern (Table 4, Model 3), whereas those who had noticed no changes in wellbeing, as well as those who received sufficient information, were less likely to report such an increase. Those who considered the visiting restrictions to be the wrong solution were much more likely than those who agreed with it to report markedly increased concern.

The Nagelkerke *R*^2^ was highest in Model 3 (0.46) (Table 4), indicating that this model enabled us to capture much more of the variation in the dependent variable than did Models 1 (0.09) and 2 (0.16).

## Discussion

Our aim was to explore the factors associated with experienced concern among the FMs of nursing home residents during the visiting restrictions in spring 2020. Increased concern about the wellbeing of a close relative living in a nursing home was extremely common (79%), and almost half of the respondents reported a deterioration in their own wellbeing on account of this concern. However, it is worth noting that 49 percent of respondents failed to report any decrease in their wellbeing. Relatives’ wellbeing obviously depends on a variety of factors. However, within the framework of this study, our multivariate analyses revealed that the factors associated with a notable increase in concern were primarily connected with the visiting ban: the adequacy of contact and information, observations of changes in the wellbeing of relatives, and doubts over the appropriateness of the visiting restriction. We are unable to draw conclusions about causality based on the findings from these cross-sectional analyses. Thus, we can only state that those who considered the visiting restrictions to be the wrong solution were more likely to report markedly increased concern: whether this concern occurred before or after the arising of such an opinion cannot be assessed. By contrast, the associations between respondent background variables and concern were generally weaker.

In line with previous research [42], perceived (in)adequacy of information provided by the nursing home was associated with a notable increase in concern among respondents. However, the provision of information did not prevent feelings of concern: approximately two in five respondents who had received sufficient information reported some increase in concern. Thus, it appears that, although active information delivery probably decreases fear and worry among the FMs of nursing-home residents, pandemics such as COVID-19 represent such an unexpected and violent threat that no information delivery can eliminate this concern entirely. Previous viral epidemics, such as the case of the norovirus, have certainly resulted in similar visiting restrictions in nursing homes, but the COVID-19 situation has been exceptional in both its duration and geographical distribution. Our findings demonstrate that people are aware of both the uncontrollable and unpredictable nature of such diseases and the way viruses spread in facilities offering long-term care. It is worth noting, however, that the satisfaction or dissatisfaction with information provision expressed by the respondents cannot be taken to directly represent their assessment of communication from nursing homes alone. It is plausible to assume that when answering the question many considered information delivery in a wider sense, including that provided by ministries, national health officials, and hospital districts, whose roles may have become mixed in the survey responses.

Although increased concern was largely attributable to the respondents’ experiences during the early COVID-19 pandemic, rather than to background variables, some pre-COVID-19 behaviors were strongly connected to their level of concern. For example, concern was clearly linked to the frequency of visits to the nursing home: those who had visited almost every day before the pandemic reported extremely high levels of concern compared to those who had visited less frequently. The vast majority of both our entire sample (88%) and frequent visitors (92%) were female FMs of residents, which is consistent with previous research [25, 26]. Distance from the home did not explain the differences in reported concern, however. Moreover, it is possible that extremely frequent visitors to nursing homes were worried about their relatives before the outbreak of the pandemic [28, 30]. As noted above, many FMs take an active part in caring for relatives, which is attributable in part to their belief that their own involvement contributes to the quality of the care provided in the nursing home [26, 29]. Frequent visitors may feel personally responsible for the care [6] and thus also feel increased concern when they are prevented from visiting. As previous COVID-research demonstrates [44], the loss of such a caregiving role proved a major source of distress to family members during the visiting ban. It is also worth noting that Finnish nursing homes seldom offer distinct visiting hours; rather, FMs may visit when they choose. Thus, the contrast between the period of the visiting ban and pre-pandemic times was stark and may, for its part, explain the extent of FMs concern (79%) in this study.

One potential reason for concern rooted in pre-COVID-19 times is decreased public confidence in elderly care in Finland, which has been widely reported as a “care crisis” in news media. Several cases of inadequate eldercare came to light in 2018, and many nursing homes managed by private enterprises were either closed or taken over by municipal actors. As Szebehely and Meagher [35] indicate, elderly care has been more rapidly privatized in Finland and Sweden than in other Nordic countries, resulting in a poorer level of universalism in services. In addition, Finnish older persons receive both long-term care and home care less frequently than do older persons in other Nordic countries; moreover, Finnish national expenditure on elderly services is the lowest in the region [46]. This low funding level has resulted, for example, in lower staffing ratios in Finnish nursing homes [47], which inevitably affects nursing staff’s ability to remain in active contact with residents’ FMs. The care crisis of 2018 showed that relatives’ mistrust in the quality of care in nursing homes highlighted in previous research [26, 28–30] was not misplaced. Thus, it is plausible that the latest Finnish care crisis is echoed in our research findings.

## Strengths and limitations

The respondents were individuals with a close relative living in a nursing home during the data-collection period. We found no information available on this target population with which to compare our sample. However, we were able to reach people from different age groups. In line with previous research findings [26, 27], the largest group comprised the 50–59-year-old daughters of nursing-home residents. Many lived near the nursing home. Finland is a sparsely populated country with long distances between residential areas, and it may be that those who were unable to visit their relative’s nursing home as frequently were less interested in responding to the survey.

The invitation to participate in the online survey was not targeted at any special sample and was distributed on Facebook, Twitter, and via the webpages and bulletins of two major Finnish non-profit organizations advancing wellbeing among older people. Thus, the oldest FMs may be underrepresented due to the online form of the survey.

Although our data contained many neutral responses, it is also possible those individuals experiencing concern were more likely to participate in the survey. Thus, our results on the frequency of concern may contain some selection bias; however, this is unlikely to impact our findings on the associated factors. Our primary goal in this study was to explore factors that could explain an increase in concern among FMs with a close relative living in a nursing home rather than to describe levels of concern in the total population with relatives residing in such institutions.

## Conclusions

We found increased concern about the wellbeing of a close relative living in a nursing home to be extremely common—a result which mirrors previous research [42–44]. However, our exploratory research adds to knowledge on the precise factors associated with experienced concern among nursing home residents’ FMs. In exceptional circumstances such as the COVID-19 related visiting ban, concern easily rises to a level that threatens the wellbeing of FMs, and almost half of the respondents reported a deterioration in their own wellbeing attributable to such concern. The take-home message of this study is that although active information provision might not prevent FMs from worrying, it certainly eases their fear and alleviates their experienced concern. It is important for FMs to receive information on the procedures used by institutions to tackle the pandemic and secure the health and wellbeing of their residents. Nonetheless, equally important to them is the provision of individualized information on how their elderly relatives are coping emotionally with such an exceptional situation. Such information would probably alleviate some of the concern that FMs currently experience. Providing FMs with more individualized information would require more eldercare resources. Our findings indicate that this would be money well spent.

## Data Availability

The datasets used and/or analyzed during the current study are available from the corresponding author on reasonable request.
